# Factors affecting the behavior outcomes on post-partum intrauterine contraceptive device uptake and continuation in Nepal: a qualitative study

**DOI:** 10.1186/s12884-019-2310-y

**Published:** 2019-05-02

**Authors:** Kusum Thapa, Rolina Dhital, Sameena Rajbhandari, Shreedhar Acharya, Sangeeta Mishra, Sunil Mani Pokhrel, Saroja Pande, Emily-Ann Tunnacliffe, Anita Makins

**Affiliations:** 1 Nepal Society of Obstetricians and Gynaecologists, Paropakar Maternity and Women’s Hospital, Thapathali, Kathmandu, GPO: 23700 Nepal; 2 Department of Obstetrics and Gynecology, Lumbini Zonal Hospital, Butwal, Nepal; 3 Department of Obstetrics and Gynecology, Koshi Zonal Hospital, Biratnagar, Nepal; 4 Department of Obstetrics and Gynecology, Bharatpur Hospital, Bharatpur, Nepal; 5 International Federation of Obstetrics and Gynaecology, London, UK

**Keywords:** PPIUD, Uptake, Continuation, Post-partum mothers, Theory of planned behavior, Nepal

## Abstract

**Background:**

The use of post-partum family planning (PPFP) methods such as post-partum intrauterine device (PPIUD) in general remains low despite its benefits for the women. The reasons or factors affecting the uptake and continuation of such PPFP methods in developing countries such as Nepal remains unclear. This qualitative research aims to explore the factors affecting PPIUD uptake and continuation related behaviors among post-partum mothers within 6 weeks of childbirth in Nepal.

**Methods:**

This qualitative study was conducted through 43 in-depth interviews among post-partum mothers who delivered in 3 selected hospitals in Nepal. Data were analyzed through content analysis using the theory of planned behavior (TPB) as the theoretical framework.

**Results:**

The themes and categories were structured around the three major components of the TPB on attitude, subjective norms, and behavioral control. Majority of the women in this study, irrespective of their behavioral outcome expressed a positive attitude towards PPIUD use. However, the women who expressed an unfavorable attitude towards PPIUD influenced their behavior to not choose or discontinue PPIUD. Subjective norms such as the family, peer, and societal influences against PPIUD negatively affected the women’s intention and behavior related to PPIUD. Whereas, the positive influence of the health providers positively affected their behavior. Regarding the behavior control, women who had their own control over decisions tended to use PPIUD. However, external factors such as their husband’s preference or medical conditions also played a prominent role in preventing many to use PPIUD despite their positive intentions.

**Conclusion:**

As suggested in TPB, this study shows that multiple factors that are interlinked affected the behaviors related to uptake and continuation of PPIUD. The attitude helped in s`haping intention but did not always lead to the behavioral outcome of PPIUD uptake and continuation. Subjective norms had a strong influence on both intention and behavior. Behavior control belief also had an important role in the outcome with respect to PPIUD uptake and continuation. Thus, a more layered, multidimensional and interlinked intervention is necessary to bring positive behavior changes related to PPIUD.

**Electronic supplementary material:**

The online version of this article (10.1186/s12884-019-2310-y) contains supplementary material, which is available to authorized users.

## Background

The use of modern contraceptives in general remains low in low- and middle-income countries (LMICs) as compared to the high-income countries [1, 2]. Evidence suggests that myths and misconceptions regarding long acting modern contraceptives such as intrauterine contraceptive devices (IUDs) have attributed to the low usage of IUDs in LMICs [1]. Studies from African countries such as Ghana and Burundi have identified lack of adequate knowledge among users, socio-cultural influences, health providers’ influences, and availability of the IUD as key barriers of uptake and continuation of IUD among users [3, 4].

LMICs in South Asia such as Nepal are no exception to the challenge of low uptake and continuation of IUDs including the ones used in the immediate post-partum period [5]. Post-partum intrauterine contraceptive device (PPIUD) is an effective and affordable long-acting post-partum family planning (PPFP) method which can be used immediately after childbirth within 48 h of post-partum period. It is known to be safe and has broad eligibility criteria for post-partum mothers [6, 7]. PPFP such as PPIUD was first introduced in Nepal between 2008 and 2009 [8]. Despite the decade-long effort, the country still lacks nationally representative data on the usage of PPIUD separately. Moreover, the overall usage of IUD remains as low as 1.4% in the country [9].

Since 2015, the Nepal Society of Obstetricians and Gynaecologists (NESOG) carried out the initiative on institutionalizing PPFP services in 6 referral facilities through immediate long-acting method such as PPIUD in Nepal [10, 11]. The initiative was supported by the International Federation of Gynaecology and Obstetrics (FIGO). The major interventions of the initiative includes training of the doctors and nurses working in the maternity units of the implementing facilities to provide timely and quality PPFP services to the women who give child birth in the facilities [11]. The interventions also include advocacy with the key family planning stakeholders in Nepal, and implementation of behavior change communication (BCC) strategies through counselling of women on PPFP as part of antenatal care (ANC) and immediate post-partum care with the use of BCC materials such as leaflets, posters and videos on benefits of PPFP including PPIUD use.

NESOG has been working closely with the government line agencies with the aim to sustain the progress and scale up the program nationwide. The initiative has been able to improve the acceptance of PPIUD among post-partum mothers above the national average rate in the 6 facilities involved. However, the uptake still remains low and discontinuation rate among the users persists. Previous study from the same initiative suggested that the total uptake of PPIUD was around 3% of 70,098 total deliveries and 10% of the 20,679 women who had been counselled on PPIUD in the 6 implementing facilities in Nepal between 2016 and 2017 [10].

Factors behind the behavioral outcomes related to uptake and continuation among the users for PPIUD are less understood. Understanding the underlying behavioral factors that has been directly or indirectly affecting the uptake and continuation could help improve the PPIUD program implementation strategies in Nepal and other LMICs with similar contexts. Therefore, this study intends to explore the factors affecting these behavioral outcomes using the theory of planned behavior (TPB) as a theoretical framework [12].

### Theory of planned behavior (TPB)

TPB has been widely used in health research to predict and explain a wide range of health behaviors including health services utilization, breastfeeding, substance use, condom use, and fertility intention [13–17].

TPB states that behavioral outcomes depend on both intention and behavioral control. It distinguishes between the three types of beliefs - attitude, subjective norms, and behavioral control. Attitude is related to favorable or unfavorable perceptions towards behavior [12]. The subjective norms deal with how the opinions of others shape an individual’s intention [10]. Behavioral control results in the actual control of the behavior that could be internal or external control which brings out an individual’s ability to decide and take action [12].

According to TPB, the combination of attitude, subjective norms and behavior control would lead to the intention which subsequently leads to a behavior outcome. TPB provides a more holistic perspective towards behavior change and suggests that improving knowledge alone or focussing on one dimension does not change a behavior [12]. Application of TPB could provide an in-depth understanding to identify gaps and design more effective interventions to improve PPIUD uptake and continuation.

## Methods

### Study setting

This qualitative study is part of a larger parent study conducted between March 2017 and June 2018. The aim of the parent study is to assess the institutionalization process of the initiative and examine the trends of PPFP counseling coverage, and PPIUD uptake and continuation in the implementing facilities. The quantitative baseline data is collected from all the mothers who had given birth in the selected facilities. A part of the quantitative study has been published recently in a larger multi-country study [10]. The data collection for this qualitative study was conducted in April 2018 in three major government referral hospitals across Nepal which were implementing PPIUD initiative. The facilities included Lumbini Zonal Hospital, Koshi Zonal Hospital and Bharatpur Hospital representing three different regions of Nepal with high obstetric caseloads of around 10,000 deliveries per year for each.

### Participants

The participants of this study were the post-partum mothers who had given birth in the selected facilities. The eligibility criteria included the mothers who had participated in the parent study during their immediate post-partum period, were at 6-weeks post-partum period and residing within the same district of the facilities. The mothers also represented four distinct PPFP related behavior outcomes which included continued PPIUD user, discontinued PPIUD user, choosing other PPFP methods, or did not choose any method.

### Data collection

**Fig. 1:**
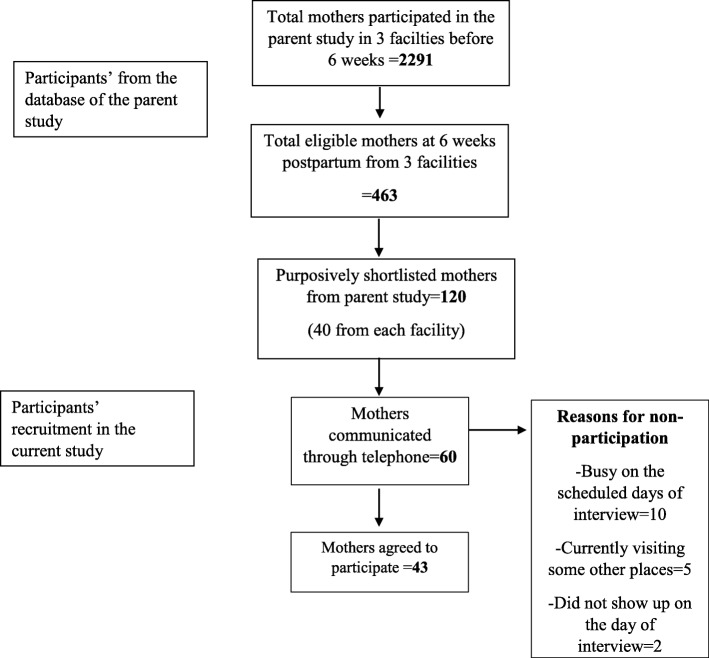
Selection of the participants from the parent study

The research team first identified 2291 mothers who had given birth and participated in the parent study from the three facilities 6 weeks before the scheduled interview dates of the current study. From the database, the team then shortlisted 463 mothers representing different PPFP related behaviors and different age groups. The team then selected 120 mothers (40 from each facility) purposively based on their address that was within the same district as that of the facilities and the completeness of their responses in the parent study. The data collection officers from each facility then tried to contact these women through the telephone. However, not all women had their own phone or were not reachable. The research team intended to recruit the women until the information reached the point of saturation for each type of PPFP related behavior in each facility. Thus, the women were contacted further and invited to participate in the following days if the information didn’t reach the point of saturation or if the invited mothers didn’t show up on the scheduled day of the interview. As a result, the data collection officers communicated with 60 mothers in total to confirm their current status related to PPFP behavior outcomes and invited them to participate in the study. However, 15 mothers refused to participate citing their unavailability on the scheduled days of the interview and 2 mothers didn’t show up on the day of the interview. In total, 43 women participated in this qualitative study (Fig. 1).

The data was collected through in-depth interviews among the women in each facility until the information reached a point of saturation for each PPFP related behavior (Additional files 1, 2, 3 and 4). The interviews were conducted in PPIUD initiative office within each facility in a quiet and secure setup. All the in-depth interviews were taken by the researcher (RD) with the support from the researchers (SA, SM, SMP) and data collection officers in each facility. All the interviews were recorded with an audio record and the field notes were taken.

### Data analysis

The audio records and field notes of all interviews were taken in Nepali language which were then translated into English language during the process of transcription by RD and the translation was reviewed by KT and SR. The transcribed information in English language was then entered into Dedoose software version 8.0.42 for data analysis. Data analysis was done through thematic content analysis using TPB as a theoretical framework [12]. The categories were identified by the researchers (KT, RD and SR) from the coding of the transcripts which were then fitted into three major themes of TPB. The analysed themes and categories were then shared with other authors (SA, SM, SMP, SP, ET and AM) for their review. Anonymous original quotes that reflected real opinions of the respondents were chosen to give more insight.

### Ethical considerations

Ethical approval was obtained from Nepal Health Research Council. The written informed consent were collected from all the participants before the interview. The participation was voluntary and confidentiality was maintained.

## Results

### Characteristics of the participants

In total, 43 women who had given birth in the selected facilities participated in this study. (Table 1) The women included continued and discontinued PPIUD users and women who preferred other PPFP methods or no methods at all.

**Table 1:** PPFP related characteristics of women participating in the study from each facility

	Facility			
PPFP behaviors	Facility 1	Facility 2	Facility 3	Total
Continued PPIUD	5	4	3	12
Discontinued PPIUD	2	3	3	8
Other PPFP methods	2	3	4	9
No method	5	5	4	14
Total	14	15	14	43

All the women who participated in this study were currently married. Among the women interviewed, the majority of them were in age group between 20 to 29 years followed by the women between 30 to 39 years of age and the least between the age of 18 and 19 years. More women in their twenties had just given birth for the first time, whereas more women in their thirties were multipara. All the adolescents interviewed had given birth just for the first time. (Table 2).

**Table 2:** Characteristics of women based on parity and age

Age	Primi-para	Multi-para	Total
Adolescent (18–19) years	6	0	6
20–29 years	16	8	24
30–39 years	1	12	13

### Themes and categories

The findings are structured around three major themes- attitude, subjective norms and behavioral control. (Table 3) The first theme on attitude was divided into perceived favorable and unfavorable outcomes that dealt with how the women perceived the use of PPIUD. The second theme on subjective norms represented various influences from the family, peers, society, and health providers in shaping their PPIUD related intention and behaviors. The third theme of behavioral control focussed on internal and external control. The original quotes of the women represents different age groups (adolescents, and women between the age groups 20–29 years and 30–39 years) and parities (primipara and multipara).

1. Attitude

**Table 3:** Themes and categories of the study

	Themes	Categories
1.	Attitude	Perceived favorable outcomes
		Perceived unfavorable outcomes
2.	Subjective norms	Family’s influence
		Peer’s influence
		Society’s influence
		Health provider’ influence
3.	Perceived behavioral control	Internal control
		External control

It has been suggested that intentions of the individual largely reflects personal attitudes towards the favourability of a behavior. However, all positive attitudes do not necessarily lead to positive behaviors [12].

### Perceived favorable outcomes

Majority of the women in this study, irrespective of their behavioral outcome expressed a positive attitude towards PPIUD use.

*“PPIUD is a less painful method as compared to depot which is injected into the arms every 3 months. PPIUD also works for many years so I felt it was a good option for me*.” (20–29 years, multipara, PPIUD user)

“*I married too soon and had a child really soon as well. But I wanted to delay my next pregnancy to complete my education. I thought PPIUD would be useful for me but I had heavy vaginal bleeding after using it and the doctor advised to remove it*” (Adolescent, Primipara, Discontinued PPIUD user)

“*I understood the benefits of PPIUD and think it is a good method. But my husband is away so I decided not to use any method*.” (20–29 years, Primipara, No methods)

“*I understood about PPIUD and it is good for those who wish to use it. I discussed with my husband about different options and decided to use depot because it is available in the health post near our home which makes it more convenient for us*” (20–29 years, Primipara, Other methods)

### Perceived unfavorable outcomes

Most of the women who expressed unfavorable perceived outcomes were PPIUD non-users or those who discontinued PPIUD.

“*Though I used PPIUD, I was just very worried about the side effects. I heard it can make you feel very weak, can cause heavy bleeding and even pierce the uterus. That is why I decided to remove it.*” (30–39 years, multipara, Discontinued PPIUD user)

“*I refused using PPIUD because I didn’t want to use a method as soon as I gave birth, I felt it would hurt me*.” (20–29 years, Primipara, No method)

2. Subjective norms

According to TPB, the individual’s intentions are largely shaped by the opinion of family, friends, or any person the individual trusts [12].

### Family influence

Strong family influence against PPIUD affected the intention and behavior of most women who were not currently using the method.

*“Everyone back home, my mother-in-law, mother, sisters, and sisters-in-law, they all said that I should have consulted them before using PPIUD. They said it is not good. I felt so worried and couldn’t bear the emotional pressure. I felt relieved after removing it.”* (20–29 years, Primipara, Discontinued PPIUD user)

A few of them who used PPIUD had a positive influence from their family.

“*During my pregnancy, I discussed the options with my husband and mother-in-law. Both suggested me to use PPIUD.*” (20–29 years, Primipara, PPIUD user)

### Peer influence

Most of the women had strong peer influence on their behavior in particular for discontinuing PPIUD and choosing other methods.

“*All my friends either use depot or pills. When I was using PPIUD, I was the only one using it which made me feel uncomfortable. I feel more comfortable now after switching to depot because I am not the only one using this method.” (*20–29 years, Primipara, Discontinued PPIUD user who switched her PPFP method)

### Social influence

Majority of the women had a strong social influence over their behavior. The major barriers included myths surrounding PPIUD use.

“*The Female Community Health Volunteer in our village asked me why I used PPIUD without consulting her. She said it has serious side effects and so many other methods were available. It just worsened my fear and I feel like removing it ever since.” (*20–29 years, Multipara, PPIUD user)

“*They said IUD can pierce our body. They said a lady from a nearby village had such complications because of IUD*.” (20–29 years, Primipara, Discontinued user)

### Health providers’ influence

Many PPIUD users agreed to use the method because they liked and understood the counselling on PPFP from the health providers.

“*I came to this hospital 4 times during my pregnancy and was counselled all the times. The health provider was so nice and explained everything very clearly. I understood different methods of PPFP and decided to choose PPIUD.*” (30–39 years, Multipara PPIUD user)

A few reflected their trust in health providers. However, they were not always counselled about PPFP.

“*I went for a check-up in a nearby health post. I always listen to their advice. They told me how to take care of myself during pregnancy and prepare for childbirth. But they never counselled about PPFP. I didn't know about PPIUD or other methods.”* (Adolescent, Primipara, No methods)

## Perceived behavioral control

### Internal control

The internal control focussed on a woman’s ability to take her own decisions irrespective of other influences or attitudes.

Many women who were currently using PPIUD reflected their own control over the decision.

“*After understanding different options I decided to use PPIUD. It is comfortable and I plan to continue using it until I decide on a permanent method”* (30–39 years, Multipara, PPIUD user)

A few of them who discontinued PPIUD or did not choose the method too reflected their own decision.

“*It was my own decision to use as well as to remove PPIUD. I just didn’t feel like continuing it from the bottom of my heart.” (*20–29 years, Multipara, Discontinued PPIUD User)

“*I understood the method well. I would have used it if my husband was around. But he is working abroad and won’t be able to come back for few years. He said I can still use it but I chose not to use it. I will think about a method when he comes back”* (20–29 years, Primipara, No method)

### External control

The external factors could be a decision made by family members on women’s behalf or complications beyond the women’s control. Most of them reflected external factors that prevented them to use PPIUD despite their own positive intentions towards it.

“*I was OK with any method. But my husband had used the condom in the past and it was his decision that we continue using a condom.”* (20–29 years, Primipara, Other methods)

*“I would have continued using PPIUD. But it was partially expelled. It wasn’t 45 days yet after my childbirth so I couldn’t reuse it immediately.”* (Adolescent, Primipara, Discontinued user)

“*I had agreed to use PPIUD but told them to ask for my husband’s opinion. My husband suggested not to use PPIUD but use depot instead. So I didn’t use it and planning to use depot*”. (Adolescent, Primipara, No methods)

## Discussion

As suggested in TPB [12], this study shows that multiple factors influence the behavioral outcomes related to uptake and continuation of PPIUD among the women. The findings highlighted the need for a multidimensional approach to improve the attitude, subjective norms and behavioral control to improve PPIUD uptake and continuation.

In this study, attitude had an influence in shaping a woman’s intention in using PPIUD. However, it also highlighted that positive attitude alone was not enough to result in actual behavior outcome. Contrary to this, a negative attitude towards PPIUD was more likely to lead to women not choosing or discontinuing PPIUD. Though the beliefs about the effect of PPIUD played a crucial role in shaping either positive or negative attitudes, other factors such as intention to have a child immediately and family member’s influence also played important roles. A study by Ajzen, on fertility intention too suggested that the reasons behind positive and negative attitudes may not entirely be the same and attitude alone may not lead to behavior [17].

As explained in TPB [12], subjective norms also played an important role in shaping one’s intention and behavioral outcomes in this study. Influences from the family and society on women’s family planning behaviors have been discussed widely [3, 4]. Similar to previous studies, myths over IUD were an important barrier that affected subjective norms [3, 4]. In this study, peer influence too affected PPIUD uptake and continuation which was related to the fear of being left alone among the peers rather than the direct effect of PPIUD. A study on PPIUD uptake among African American adolescents has also shown the influence of peers on PPIUD behavior [18].

Moreover, this study also showed that lack of timely counseling on PPFP in the peripheral facilities led to missed opportunities. Health providers play a key role to address PPFP needs in a timely fashion [19, 20]. The previous quantitative study on PPIUD initiative indicated that multiple counseling by health providers had a significant influence on the uptake of PPIUD by women [10]. Another qualitative study focussing on the training of health providers on PPFP counseling service and PPIUD insertion techniques had suggested that regular mentoring had helped in motivating the health providers on improving their services [11]. Despite the efforts, the gap on counseling exists which is partly attributed to the low health providers to patients ratio in these busy hospitals [10]. The task shifting of PPFP services such as counseling by establishing community linkages could help address the gap to some extent. The female community health volunteers (FCHV) and the peripheral health facilities are often the first points of contact for most women in Nepal [21, 22]. Involvement of FCHVs by building their capacity has proven beneficial for many health interventions in Nepal [23, 24]. The capacity building process by integrating the training packages on PPFP counseling for them into the national health system and by strengthening the local leadership to drive these training activities would be pertinent. Further, their inclusion in the BCC activities is crucial to gaining women’s trust in the family planning method.

In this study, both perceived internal and external control also played important roles in shaping the intention as well as the behavioral outcomes. Perceived behavior control is believed to have a direct influence over the behavioral outcomes irrespective of the attitude and subjective norms [12]. Internal control in this study reflected the personality attributes such as the women’s self-confidence and her internal feelings. Whereas, external controls were related to subjective norms or health complications which had no influence on a woman’s attitude or intention. It is clear from the responses that in many instances the husband is in fact in control of the chosen method of contraception. Migrant husbands are not keen to leave their wives behind with a method of contraception in situ which they do not perceive as necessary given their absence. Perhaps inclusion of partners in counselling sessions would result in a better understanding of the advantages of a one stop approach and the efficacy of PPIUD in preventing pregnancies when comparing to other methods requiring user input.

This study has certain limitations. First, this study was conducted in a small population and therefore may not be generalised to a wider population. However, as a qualitative study, this study provides an in-depth perspective of women of different age groups, parities and PPFP related behavior. Second, this study was conducted at 6 week post-partum period and does not provide a longitudinal perspective on the contraceptive choices and behaviors of the women in the long run.

Despite the limitations, the findings of this study have important program implications for stakeholders related to PPFP. The findings highlight existing gaps and will aid in designing more effective interventions through evidence based practice. Community awareness through innovative approaches using mass media and community mobilization is an effective intervention [25] which could help address attitude as well as subjective norms and behavior control. As with ANC group counselling [26], more focused group counselling on PPFP during ANC could also help in shaping positive peer influence. Moreover, involving family members such as husband or mother-in-law in the PPFP counselling could also be effective to overcome barriers surrounding subjective norms and external control [27]. Further, expansion of the institutionalization process to the peripheral facilities could help in capacity building of health providers and to reach out to more women in need of PPFP services. This would in turn widen the sphere of women using the method and hence make it more acceptable to the wider public [25].

This study is one of the few studies that have explored the factors affecting the behavior outcomes in LMICs such as Nepal. This study indicates that a more layered, multidimensional and interlinked intervention is necessary to bring out the ultimate outcome of improving the uptake and continuation of PPIUD among women in LMICs.

## Conclusion

This study identified that the multiple factors as outlined in TPB influenced the behavior related to uptake and continuation of PPIUD among women and each factor was interlinked to the others. The attitude helped in shaping intention but did not always lead to the behavioral outcome of PPIUD uptake and continuation. Subjective norms had a strong influence on both intention and behavior. Behavior control belief also had an important role in the outcome with respect to PPIUD uptake and continuation. Further studies on larger population with a longer follow up could provide a broader and longitudinal perspective.

## Additional files

Additional file 1: Interview guide for PPIUD continued user. (DOCX 30 kb)

Additional file 2: Interview guide for PPIUD discontinued user. (DOCX 31 kb)

Additional file 3: Interview guide for women who chose other PPFP methods. (DOCX 31 kb)

Additional file 4: Interview guide for women who chose no PPFP methods. (DOCX 31 kb)

## Abbreviations

ANC: Antenatal care
BCC: Behavior change communication
IUD: Intrauterine contraceptive device
LMIC: Low and middle income countries
NESOG: Nepal Society of Obstetricians and Gynecologists
PPFP: Post-partum family planning
PPIUD: Post-partum intrauterine contraceptive device
TPB: Theory of Planned Behavior
