# Supplementary material for: Factors affecting the behavior outcomes on post-partum intrauterine contraceptive device uptake and continuation in Nepal: a qualitative study
Source: BMC Pregnancy Childbirth. 2019 May 2;19:148. doi: 10.1186/s12884-019-2310-y (PMC6498647; doi:10.1186/s12884-019-2310-y)
Supplement: Supplementary file 3 — Interview guide for women who chose other PPFP methods. (DOCX 31 kb) [file 12884_2019_2310_MOESM3_ESM.docx]

| **NESOG FIGO PPIUD project**  **In-depth Interview checklist for women who chose other PPFP methods** | | |
| --- | --- | --- |
|  | **Identification** |  |
| 01 | NAME OF FACILITY_________________________________________ |  |
| 02 | RESPONDENT’s CODE __________________________________________ |  |
| 03 | LOCATION_______________________________________________________ |  |
| 04 | NAME OF INTERVIEWER__________________________________________ |  |
| 05  06 | DATE INTERVIEWED.....................................................................................  Time Interview started: Hour: ___ Minute: ___ | DAY MONTH YEAR |

**Factors affecting the behavior outcomes on post-partum intrauterine contraceptive device uptake and continuation in Nepal: a qualitative study**

Interview checklist

| **Questions/ Issues** | |
| --- | --- |
| **I.** | **General background** |
|  | - What is your age? - What is your level of education? - What is your occupation? - Could you tell us a bit more about the family you were born into? - How your parents were like when you were growing up? (e.g were they too strict? Too conservative?) - How is your relationship with your parents now? - How many siblings do you have? - How is your relationship with them now? |
| **II.** | **Marriage and the family** |
|  | - When did you get married? - How old were you when you got married? - How did you first meet your husband? - How did you get married? (*e.g was it a love marriage? Arranged marriage? Was it a forced marriage?)* - What is your husband’s occupation? - What is your husband’s level of education? - Whom do you currently live with? (*e.g husband, mother in-law, father in-law etc*.) - Who is the head of the household or takes the decision in your family? - How is your relationship with your husband? - How is your relationship with your in-laws? - How is your relationship with rest of the family? - What changes has marriage bring in your life? - How much of authority do you think you have in your family? (*e.g to decide what is best for yourself and your family*) |
| **III.** | **Family planning** |
|  | - How many children do you have? - Were your last pregnancy and previous pregnancies (if any) planned? - What changes did having a child/children bring in your life? - Do you have plans to have more children?   -If yes, why?  -If no, why?   - Did you ever discuss family planning with your husband?   -If not, why didn’t you discuss about family planning?  -If yes, why did you discuss about family planning?  -What did you discuss about family planning?   - Did you discuss about family planning with anybody else?   -If yes, who did you discuss with?  -What did you discuss about?   - Have you ever used any FP method before your last pregnancy?   -If yes, what did you use?  -What made you use the contraceptive?  -How did you use the contraceptive?  -Who decided that you should use the contraceptive?  -If No, why didn’t you use?   - Do you wish to have more child/children in the future?   -If yes, why?  -If no, why? |
| **IV.** | **Experience with childbirth** |
|  | - How was the experience of your last childbirth in the hospital? - Was it a vaginal birth or by CS? - How long was the labour? - How did you feel after you delivered? - How did you feel when you first saw your new born? - Did you wish for a specific sex for your new born?   -If yes, why?  -If no, why?   - Were you happy with the sex of your new born?   -If yes, why?  -If no, why?   - How long did you stay in the hospital? |
| **V.** | **Experience with current FP method** |
|  | - What made you choose the FP method you ware currently using after you delivered your last child? - Who provided the counselling to you? (e.g doctor, nurse, counsellor etc) - How many time were you counselled? - When were you counselled each time? (at which stages of pregnancy?) - How was your experience with the health provider counselling you on PPFP? - Who made the decision that you should use FP method you are currently using? (e.g yourself, husband, mother-in law etc. - Was anybody else from your community/ neighbourhood involved in the decision? (such as FCHVs, friends etc) - How was your experience with the health provider providing FP method you are currently using? - How long do you want to continue using this method? And Why? - Is there any message or advice you want to give to health workers who provided PPFP service to you? |

Thank you very much for your help.

Time interview ended: ………..hour ………minute
